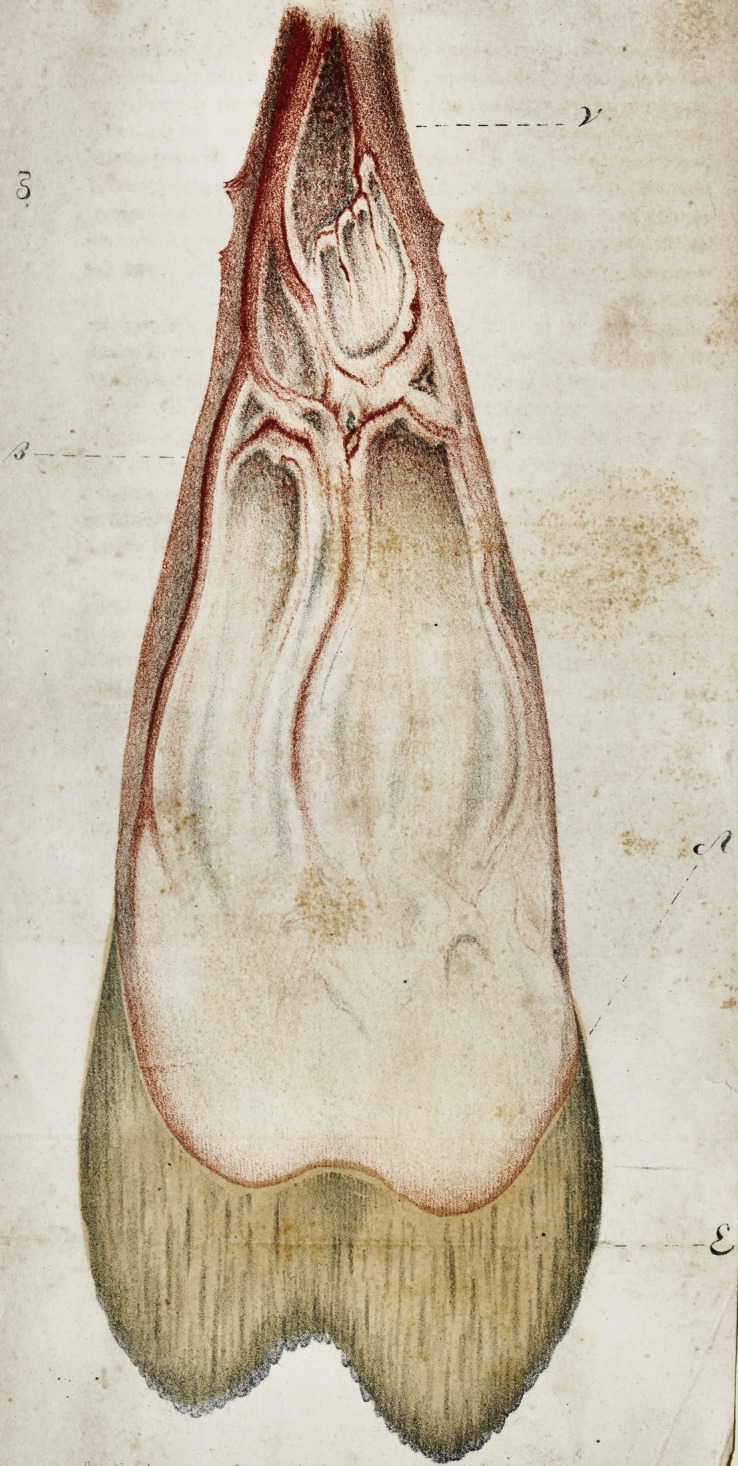# Letter from Mr. C. Brown, to the Baltimore Editor

**Published:** 1841-09

**Authors:** Charles Brown


### ARTICLE VII.

### *Letter from* MR. C. BROWN, *to the Baltimore Editor.*

HONOURED AND DEAR SIR: *Woolwich, Kent, England.*

THE observation of the erudite and acute reasoner, President Edwards, who, "though dead, yet speaketh," that *most of the great things that have been done in the world, the great revolutions that have been accomplished* in the kingdoms and empires of the earth, have been chiefly owing to the influence and power of *zeal and resolution,* is a proposition, the truth of which, I am persuaded, will, eventually, be most satisfactorily demonstrated, in results worthy the exertions now making by yourself and colleagues, to emancipate our profession from its present degraded condition. And, although, all the benefits that could be wished, may not be achieved immediately, nor, perhaps, in the life-time of those who are now so laudably striving to effect it; still, every member of our body who really feels the elevation of moral character, and the advancement of scientific attainments in the majority of prac-

From a drawing furnished by

Mr Chas Brown Surgeon Dentist Woolwich, England.

For the American Journal & Library of Dental Science

Lithogr & printed in Colours by E. Weber & Co Baltimore titioners, to be a desideratum; or, who consider it in a more comprehensive sense, as involving, in no small degree, the welfare and happiness of community, and some of our dearest interests, as honourable members of society, and the interests of our posterity (especially to such of us who have children coming forward in our practice) must consider their sincere congratulation, and grateful acknowledgments, are due to our brethren of the United States, for their *zeal, and for their resolution,* and will be anxious to encourage and promote, as far as ability may serve, so important an object.

Influenced by this feeling, I, with much pleasure, forward to you the accompanying drawing, with the hope that it will be of some assistance to you in your demonstrations, and may, in some degree, facilitate further inquiries into the physiology of the teeth; and, if thought worthy of lithographing, it may produce a trifling profit, and so made available to the support of "the Journal." The drawing, as you will doubtless perceive, is from a microscopic view of the internal membrane of a superior bicuspis, the history of which I annex. For the accuracy of the delineation, I can avouch, having drawn it myself, (and another which I gave to our esteemed and justly popular fellow practitioner, J. Bell, Esq., F. R. S.) direct from the microscope.

That much honour and satisfaction may be the reward of yourself and fellow-labourers, is the sincere wish of yours, dear sir,

Very respectfully, CHARLES BROWN.

EXPLANATION OF THE PLATE.

The tooth was removed in order to relieve a crowded denture. The patient, a young artillery officer, at the time of its extraction, *was very heated by a hasty run* to my house. He is, also, of *very plethoric habit.*

At the expiration of about six weeks, the tooth was carefully split in a vice, when I had the satisfaction to obtain the very beautiful specimen, from which the drawing is taken. The membrane itself has now lost its fainter colours, but the principal vessel $\beta$ is still perfect and distinct. By reason of the difficulty of detaching it, the membrane, from the foramen in apex of the fang, is ruptured and imperfect at and above the line $\gamma$. The vessels appear to concentrate and terminate at $\lambda$, not entering the substance at $\varepsilon$ below. The parts marked $\zeta$ are novel to me, but they exist in the specimen, and appear to occupy a position similar to the byssus of a bivalve shell-fish.

## Figures and Tables

**Figure f1:**